# Hypoxia and hypotension in patients intubated by physician staffed helicopter emergency medical services - a prospective observational multi-centre study

**DOI:** 10.1186/s12873-017-0134-5

**Published:** 2017-07-11

**Authors:** Geir Arne Sunde, Mårten Sandberg, Richard Lyon, Knut Fredriksen, Brian Burns, Karl Ove Hufthammer, Jo Røislien, Akos Soti, Helena Jäntti, David Lockey, Jon-Kenneth Heltne, Stephen J. M. Sollid

**Affiliations:** 1 Norwegian Air Ambulance Foundation, Drøbak, Norway; 2 Department of Anaesthesia and Intensive Care, Haukeland University Hospital, Bergen, Norway; 3 Department of Health Sciences, University of Stavanger, Stavanger, Norway; 4 Air Ambulance Department, Oslo University Hospital, Oslo, Norway; 5 Faculty of Medicine, University of Oslo, Oslo, Norway; 6 University of Surrey, Guildford, UK; 7 Kent, Surrey & Sussex Air Ambulance Trust, Marden, UK; 8 UiT - The Arctic University of Norway, Tromsø, Norway; 9 The University Hospital of North Norway, Tromsø, Norway; 10 Sydney HEMS, NSW Ambulance, Sydney, Australia; 11 Sydney Medical School, University of Sydney, Sydney, Australia; 12 Centre for Clinical Research, Haukeland University Hospital, Bergen, Norway; 13 Hungarian Air Ambulance Nonprofit Ltd, Budaors, Hungary; 14 Centre for Pre-hospital Emergency Care, Kuopio University Hospital, Kuopio, Finland; 15 London’s Air Ambulance, Bartshealth NHS Trust, London, UK; 16 Department of Medical Sciences, University of Bergen, Bergen, Norway; 17 Norwegian Air Ambulance Foundation, Møllendalsveien 34, 5009 Bergen, Norway

**Keywords:** Physician staffed HEMS, Airway management, Intubation, Air ambulance, Helicopter emergency medical services, Advanced trauma life support, Critical care

## Abstract

**Background:**

The effective treatment of airway compromise in trauma and non-trauma patients is important. Hypoxia and hypotension are predictors of negative patient outcomes and increased mortality, and may be important quality indicators of care provided by emergency medical services. Excluding cardiac arrests, critical trauma and non-trauma patients remain the two major groups to which helicopter emergency medical services (HEMS) are dispatched. Several studies describe the impact of pre-hospital hypoxia or hypotension on trauma patients, but few studies compare this in trauma and non-trauma patients. The primary aim was to describe the incidence of pre-hospital hypoxia and hypotension in the two groups receiving pre-hospital tracheal intubation (TI) by physician-staffed HEMS.

**Methods:**

Data were collected prospectively over a 12-month period, using a uniform Utstein-style airway template. Twenty-one physician-staffed HEMS in Europe and Australia participated. We compared peripheral oxygen saturation and systolic blood pressure before and after definitive airway management. Data were analysed using Cochran–Mantel–Haenszel methods and mixed-effects models.

**Results:**

Eight hundred forty three trauma patients and 422 non-trauma patients receiving pre-hospital TI were included. Non-trauma patients had significantly lower predicted mean pre-intervention SpO_2_ compared to trauma patients. Post-intervention and admission SpO_2_ for the two groups were comparable. However, 3% in both groups were still hypoxic at admission. For hypotension, the differences between the groups were less prominent. However, 9% of trauma and 10% of non-trauma patients were still hypotensive at admission. There was no difference in short-term survival between trauma (97%) and non-trauma patients (95%). Decreased level of consciousness was the most frequent indication for TI, and was associated with increased survival to hospital (cOR 2.8; 95% CI: 1.4–5.4).

**Conclusions:**

Our results showed that non-trauma patients had a higher incidence of hypoxia before TI than trauma patients, but few were hypoxic at admission. The difference for hypotension was less prominent, but one in ten patients were still hypotensive at admission. Further investigations are needed to identify reversible causes that may be corrected to improve haemodynamics in the pre-hospital setting. We found high survival rates to hospital in both groups, suggesting that physician-staffed HEMS provide high-quality emergency airway management in trauma and non-trauma patients.

**Trial registration:**

Clinicaltrials.gov Identifier: NCT01502111. Registered 22 Desember 2011.

## Background

Pre-hospital advanced airway management including tracheal intubation (TI) has high priority in the management of critically ill patients [1–3]. Drug-assisted rapid sequence intubation (RSI) is the definitive method of securing the airways of patients who are unable to maintain patent airways or adequate ventilation [2]. However, TI in the pre-hospital setting may be challenging, with sub-optimal working conditions for critical care providers [4]. Several studies report a high incidence of unanticipated difficult airways, first TI attempt failures and complications during pre-hospital advanced airway management, comparable to emergency airway management outside the operating room [5–8]. Critically ill patients may be susceptible to hypoxia and hypotension in conjunction with emergency anaesthesia and airway management [9–11]. The full range of optimal emergency airway management requires an experienced and trained provider to manage it, and hospital-level care to patients in the field is often provided by physician-staffed helicopter emergency medical services (HEMS) [12–14].

Pre-hospital hypoxia and hypotension are predictors of negative patient outcomes and increased in-hospital mortality in non-cardiac arrest patients, and avoidance or mitigation of hypoxia and hypotension may be considered important measures of quality of care provided by the emergency medical services (EMS) [15–17]. Sadly, core data on physiological responses and how they relate to pre-hospital TI are inconsistently reported. Standardised data from pre-hospital airway management could improve our knowledge about the challenges of hypoxia and hypotension in TI [18–20].

The target group of this multi-centre study were non-cardiac arrest patients requiring pre-hospital TI by physician-staffed HEMS. By excluding out-of-hospital cardiac arrests, critical trauma and non-trauma patients are the major groups to which HEMS are dispatched [21]. Several studies describe the impact of pre-hospital hypoxia or hypotension on trauma patients but few studies compare this to the impact hypoxia and hypotension has on non-trauma patients needing pre-hospital TI. This knowledge could be important for how the two groups are handled in pre-hospital care [15, 22]. The primary aim of our study was to describe the incidence of pre-hospital hypoxia and hypotension in the two groups. Secondarily, we wanted to assess whether survival to hospital differed between trauma and non-trauma patients.

## Methods

### Study design and setting

This prospective multi-centre study collected uniform data on advanced pre-hospital airway management from 21 HEMS in Australia, England, Finland, Hungary, Norway and Switzerland, to analyse differences between trauma and non-trauma patients requiring TI in the field. To include the full range of resuscitative interventions on-scene, only physician-staffed HEMS participated. Necessary ethical and institutional approvals were acquired prior to patient enrolment.

### Participants

Trauma and non-trauma patients requiring pre-hospital TI on primary missions were included. Primary out-of-hospital cardiac arrests were excluded. Airway management and RSI protocols were part of local standard operating procedures. Service-specific anaesthetic agents, sedatives, analgesics and neuromuscular blocking agents were used to facilitate TI.

### Data collection

Data collections lasted for 1 year for the majority of centres, commencing on the 1st of January 2012 for the majority of centres and concluded on the 15th of March 2013 for the last centre. Two centres, Kent Surrey Sussex HEMS (England) and REGA-Basel (Switzerland), participated for 9 and 6 months, respectively. Prospective airway data were collected according to dataset definitions described in the Utstein style template [23]. Survival data was available only for the pre-hospital phase, as in-hospital follow-up was beyond the scope of this study. Data regarding airway management success rates and complications have previously been published [24].

### Variables

Patient demographics were described by category (trauma or non-trauma), age, sex, and indications for TI. Burns and strangulation were classified as trauma in the template, while drownings and asphyxia were classified as non-trauma. We compared lowest oxygen saturation (SpO_2_) and systolic blood pressure (SBP) before and after completion of TI as described in the airway template. An intubation attempt was defined as attempted laryngoscopy with the intent to intubate. Hypoxia was defined as SpO_2_ < 90% and hypotension as SBP < 90 mmHg. S_P_O_2_ and SBP were measured as first value recorded on scene and first value recorded after finalised airway management. Glasgow Coma Score (GCS) and pre-intervention comorbidity (American Society of Anesthesiologists Physical Status (ASA-PS)) were recorded. The variables are defined in the airway template paper [23].

### Statistical analysis

Categorical demographical variables are presented as counts and percentages. The multi-centre nature of the study introduces an internal clustering in the data, and the statistical methods that take this into account have thus been applied [25]. We were mainly interested in the intra-site association between the recorded variables. For binary outcomes we applied Cochran–Mantel–Haenszel methods to estimate conditional odds ratios (cOR) and corresponding 95% confidence intervals (CIs), while for continuous outcomes we used mixed-effects models with random intercepts for sites for single-value outcomes and for sites and patients for longitudinal data. Results from these models are presented as predicted means. The predicted mean is an estimate of the mean for a patient from a ‘typical’ site, i.e. with the random effect(s) set to zero, and can roughly be interpreted as a ‘mean of means’ estimate. Marginal estimates are also presented. The amount of missing data for the main outcomes was generally low, and we have therefore performed complete-case analyses throughout.

We used IBM SPSS Statistics version 21.0 (IBM Corp., Armonk, New York) for storing and preparing the data for statistical analysis and R version 3.2.1 and 3.2.3 (R Foundation for Statistical Computing, Vienna, Austria) for performing the analyses [26]. For fitting the mixed-effects models, we used the R packages ‘nlme’ version 3.1–123 and ‘lme4’ version 1.1–10.

## Results

### Demographics

**Fig. 1:**
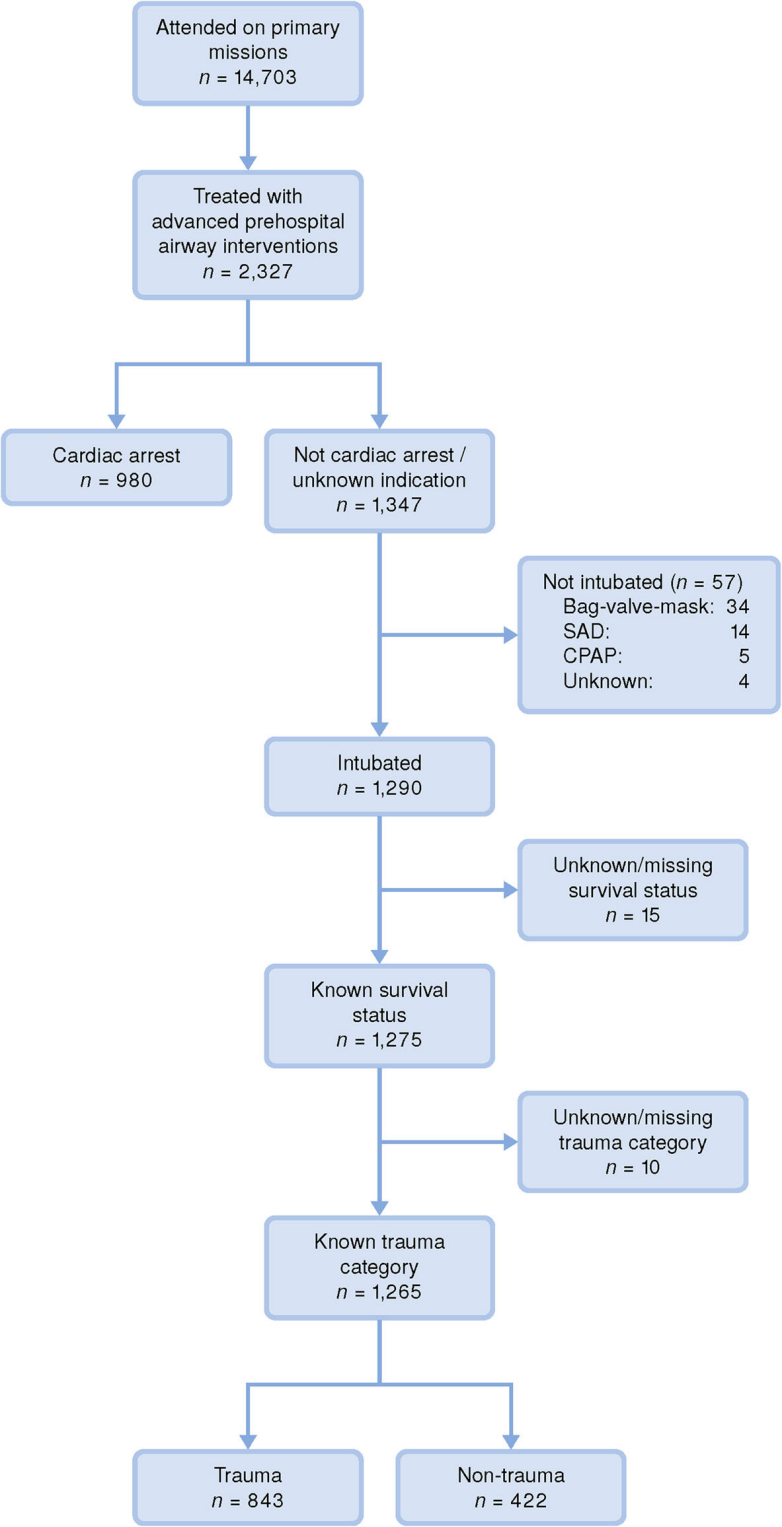
Study population flow chart. Flow chart of study population. One thousand two hundred sixty five non-cardiac arrest patients that received pre-hospital tracheal intubation were included

Overall, 2327 (16%) of patients attended required advanced airway interventions during the study period. We included 1265 patients receiving pre-hospital TI in the analysis. Of these, 843 were trauma patients and 422 non-trauma patients. Patients handled with bag-valve-mask ventilation (BVM), supraglottic airway devices (SAD) or continous positive airway pressure (CPAP) instead of TI and patients with missing data relative to airway management, short-term survival status or trauma categories were excluded from the analysis (Fig. 1). Patient characteristics, indication for TI and number of attempts are summarised in Table 1. Decreased level of consciousness was the most frequent indication recorded in both trauma (61%) and non-trauma patients (69%). Emergency surgical airway was done in two trauma patients (0.2%), one after failed primary TI and one was a primary surgical airway. The proportion of males did not differ significantly between trauma and non-trauma patients (cOR: 1.27; 95% CI: 0.94–1.73). There was a large difference in predicted mean age between trauma and non-trauma patients (43 and 54 years; CI of difference: 8–14 years). Non-trauma patients had a predicted mean initial GCS of 2.1 points lower (95% CI: 1.6–2.6) than trauma patients (predicted means 5.6 and 7.6, respectively).

**Table 1:** Marginal/crude patient characteristics

Patient category	Trauma		Non-trauma		All patients		*P*-value
	*n*	%	*n*	%	*n*	%	
Patients	843	100%	422	100%	1265	100%	
Age							< 0.001
0–5 years	19	2%	22	5%	41	3%	
6–14 years	36	4%	9	2%	45	4%	
15–29 years	227	27%	38	9%	265	21%	
30–49 years	276	33%	83	20%	359	29%	
50–69 years	185	22%	151	37%	336	27%	
> 70 years	83	10%	109	26%	192	16%	
*Missing data*	*17*	*2%*	*10*	*2%*	*27*	*2%*	
Sex							< 0.001
Male	622	74%	266	64%	888	70%	
*Missing data*	*1*	*0%*	*3*	*1%*	*4*	*0%*	
Comorbidity (ASA-PS)							< 0.001
ASA class 1	500	67%	100	26%	600	53%	
ASA class 2	188	25%	146	38%	334	29%	
ASA class 3	58	8%	120	31%	178	16%	
ASA class 4	4	1%	19	5%	23	2%	
ASA class 5	0	0%	4	1%	4	0%	
*Missing data*	*90*	*11%*	*29*	*7%*	*119*	*9%*	
Indication for pre-hospital TI							< 0.001
Decreased consciousness	510	61%	277	69%	787	64%	
Ineffective ventilation	69	8%	58	14%	127	10%	
Combative or uncooperative	93	11%	9	2%	102	8%	
Impending airway obstruction	68	8%	12	3%	80	6%	
Hypoxia	26	3%	25	6%	51	4%	
Relief of pain or distress	44	5%	4	1%	48	4%	
Existing airway obstruction	13	2%	12	3%	25	2%	
Other	8	1%	6	1%	14	1%	
*Missing data*	*12*	*1%*	*19*	*5%*	*31*	*2%*	
Attempts at pre-hospital TI							< 0.001
One attempt	772	92%	360	86%	1132	90%	
Multiple attempts	68	8%	60	14%	128	10%	
*Missing data*	*3*	*0%*	*2*	*0%*	*5*	*0%*	

Characteristics of patients requiring pre-hospital TI by physician-staffed HEMS, *TI* Tracheal intubation, *HEMS* Helicopter emergency medical services, *ASA-PS* American Society of Anesthesiologists Physical Status. All percentages except for the ‘Missing data’ rows are calculated based on the non-missing data

### Oxygen saturation

**Fig. 2:**
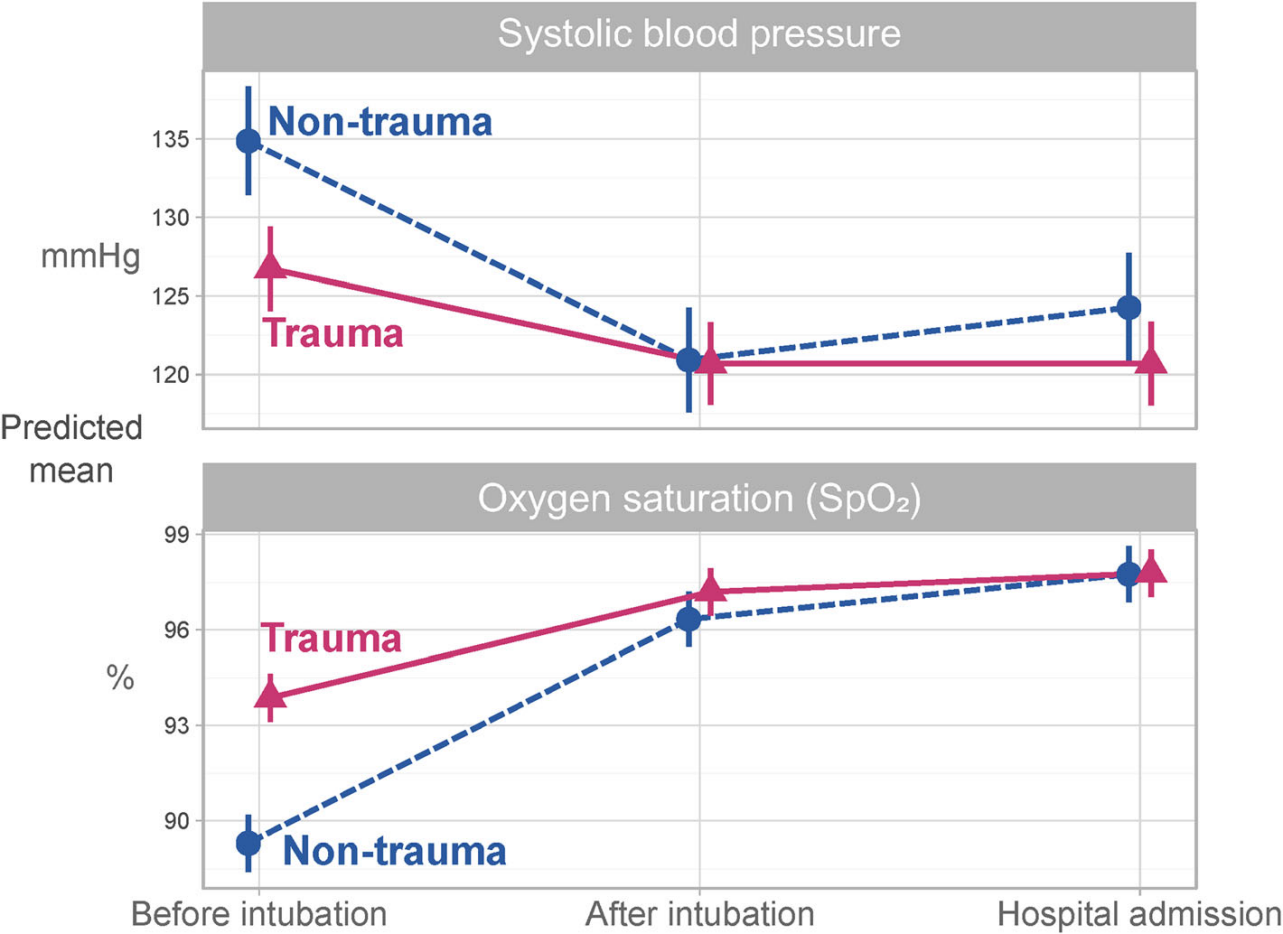
Patient vitals across airway intervention. Predicted means for patients SBP and S_P_O_2_ across airway intervention for trauma and non-trauma patients, based on linear mixed-effects models with time, trauma category and their interaction as fixed effects and random intercepts for patients and HEMS. Vertical lines show 95% pointwise confidence intervals. Non-trauma patients had a significantly lower mean S_P_O_2_, and higher mean SBP, before TI compared to trauma patients. Post-intervention and admission values for the two groups showed little difference. SBP: systolic blood pressure. S_P_O_2_: oxygen saturation. HEMS: helicopter emergency medical services

Non-trauma patients had significantly lower pre-intervention SpO_2_ (*p* < 0.001) and post-intervention SpO_2_ (*p* = 0.001) than trauma patients, predicted means 89 vs. 94%, and 96 vs. 97%, respectively (Fig. 2). Admissions mean SpO_2_ was 98% for both groups. In both groups, 3% of patients were still hypoxic at admission (Table 2). Rates of hypoxia and hypotension in patients with decreased level of consciousness are presented in Table 3.

**Table 2:** Hypotension and hypoxia rates before and after airway intervention

Patients	Trauma		Non-trauma		*P*-value^a^	All patients		Missing data	
	843	100%	442	100%	–	1265	100%	–	–
Hypoxia^b^									
Pre-intervention	126	18%	114	33%	0.01	240	23%	212	17%
Post-intervention	40	5%	31	8%	0.17	71	6%	106	8%
Admission to hospital	23	3%	12	3%	0.30	35	3%	164	13%
Hypotension^c^									
Pre-intervention	87	12%	56	15%	0.83	143	13%	157	12%
Post-intervention	100	13%	63	16%	0.97	163	14%	77	6%
Admission to hospital	68	9%	37	10%	0.12	105	9%	148	12%

The reported rates are marginal rates

^a^Based on Cochran–Mantel–Haenszel chi-squared tests

^b^Hypoxia was defined as oxygen saturation (SpO_2_) < 90%

^c^Hypotension was defined as systolic blood pressure (SBP) < 90 mmHg

**Table 3:** Hypotension and hypoxia in patients with decreased level of consciousness

Patients	Trauma		Non-trauma		*P*-value^a^	All patients		Missing data	
	510	100%	277	100%	–	787	100%	–	–
Hypoxia^b^									
Pre-intervention	63	15%	55	23%	0.23	118	18%	118	15%
Post-intervention	17	4%	13	5%	0.37	30	4%	56	7%
Admission to hospital	7	2%	3	1%	0.19	10	1%	83	11%
Hypotension^c^									
Pre-intervention	48	11%	27	11%	0.63	75	11%	88	11%
Post-intervention	55	11%	28	11%	0.44	83	11%	38	5%
Admission to hospital	34	7%	17	7%	0.20	51	7%	77	10%

The reported rates are marginal rates

^a^Based on Cochran–Mantel–Haenszel chi-squared tests

^b^Hypoxia was defined as oxygen saturation (SpO_2_) < 90%

^c^Hypotension was defined as systolic blood pressure (SBP) < 90 mmHg

### Systolic blood pressure

There was a significant difference in predicted mean pre-intervention SBP between non-trauma and trauma patients (*p* = 0.002), but not for SBP at post-intervention or at admission (Fig. 2). For non-trauma patients, there was a significant decrease (*p* < 0.001) in SBP during the airway intervention from a pre-intervention (predicted mean) SBP of 135 mmHg to post-intervention SBP 120 mmHg, and a subsequent significant increase to hospital admission SBP of 124 mmHg. For trauma patients, there was a significant (*p* < 0.001) decrease in SBP during the airway interventions from 127 mmHg to 121 mmHg, but post-intervention SBP was equal to the admission SBP of 121 mmHg. Nine percent of trauma patients and 10% of non-trauma patients were still hypotensive at admission (Table 2).

### Short-term survival

Overall, 97% of patients survived to hospital admission. There was no difference in short-term survival between trauma patients (97%) and non-trauma patients (95%) (cOR = 0.78; 95% CI: 0.34–1.68; *p* = 0.56). The large group intubated for decreased level of consciousness showed a more positive association with survival to hospital (cOR = 2.8; 95% CI: 1.4–5.4; *p* < 0.001) than other indications for TI combined (Table 1). This effect was strong for non-trauma patients (cOR = 10.7; 95% CI: 2.7–42.1; *p* = 0.001) but not present for trauma patients (cOR = 1.2; 95% CI: 0.49–2.9; *p* = 0.69).

## Discussion

### Main findings

Non-trauma patients had a significantly higher incidence of hypoxia before TI than trauma patients. Post-intervention and admission SpO_2_ for the two groups were comparable, and 3 % in both groups were still hypoxic at admission. For hypotension, the differences between the groups were less prominent, but one in ten trauma and non-trauma patients were still hypotensive at admission. There was no difference in survival to hospital between the groups studied, but patients intubated due to decreased level of consciousness showed a positive association with short-term survival than patients with other indications for TI.

### Vital signs and emergency anaesthesia

Vital signs are commonly used for initial assessment and triage of patients, both in the pre-hospital setting and emergency department [27]. Hypoxia and hypotension in the field are predictors of increased in-hospital mortality, but patients’ vital signs across airway interventions are infrequently reported [16, 18, 28]. Physiological variables like SBP and SpO_2_ may represent indications for TI, but they are also markers of success or complications following airway intervention and the level of critical care provided in the field [23, 29]. As the pathophysiology behind pre-hospital hypoxia and hypotension are diverse, it is important that these vital signs are interpreted along with other clinical variables and the mechanism of injury or illness [27, 30]. Resuscitative interventions are often initiated before the cause of hypotension or hypoxia is clearly identified [31]. The objectives of emergency anaesthesia and TI is to secure oxygenation and ventilation, but also to avoid secondary insults caused by hypoxia and hypotension to vital organs [1]. After initial resuscitation and stabilisation of the patient on-scene, pre-hospital critical care also includes a transition from anaesthesia to mobile critical care during the evacuation and transport of the patient to hospital. Although proper regular monitoring of vital signs is a priority in patients receiving emergency anaesthesia in the field, careful preparation and adequate monitoring to avoid complications can be more difficult in a pre-hospital setting than in a hospital [1, 32].

### Hypoxia

One in three non-trauma patients in our study presented with pre-intervention hypoxia, and this was significantly more frequent than for trauma patients. However, in both groups 3 % remained hypoxic at hospital admission after pre-hospital TI. Similar results have been reported from other physician-manned EMS [33, 34]. As desaturation may develop more rapidly in critically ill patients receiving emergency anaesthesia, strategies to improve preoxygenation during RSI in the field, like apnoeic oxygenation, may be important to reduce hypoxia in these patients [9, 22, 35]. We have previously published data showing a non-linear association between the patient’s age and the TI failure risk, with the highest risk for middle-aged patients and significantly lower risk for both younger and older patients [24]. Another study demonstrated significantly higher age among all patients experiencing desaturation during pre-hospital RSI, and also showed that the duration of hypoxia was significantly longer in non-trauma patients compared to trauma patients [22]. Physician-staffed EMS provide TI success rates of close to 100% with very high first pass success rates and robust RSI procedures that are effective in preventing or correcting hypoxia. Highest quality airway management can be provided before arrival in hospital [18, 36, 37].

### Hypotension

Hypotension in trauma patients is often due to hypovolemia from blood loss, while non-traumatic hypotension may be due to a variety of causes, including hypovolemic, cardiogenic, septic or neurological factors, and it may be difficult to determine the exact cause of non-traumatic hypotension in the pre-hospital setting [16, 31]. In-hospital mortality rates in non-trauma patients after pre-hospital hypotension have also shown to be high across all age groups [16, 17]. Furthermore, sustained hypotension or shock in trauma and non-trauma patients correlates with higher in-hospital mortality [17, 38]. Although clinical thresholds for hypotension related to patient outcomes, e.g. 90 mmHg for severe traumatic brain injury (TBI), has been suggested, recent studies indicate a possible linear association between pre-hospital SBP and the probability of death, suggesting that using thresholds might not be so meaningful [15, 30, 39]. Nonetheless, a limit of 90 mmHg to indicate hypotension was used in our study, in agreement with clinical guidelines and other systems handling critical ill patients [16, 31, 40]. The reductions in SBP across TI within the groups studied were statistically significant, but these changes may not be clinically significant. They may be the result of the effect of sedatives or anaesthetics perturbing physiology in critical illness or the illness itself, such as hypovolemia. Since it was not possible to standardise the medication or intravenous fluid protocols in the participating international centres, variation in use of these with hypotensive side effects can be possible confounders. The recorded SBP after TI in our study suggests that the HEMS teams have provided good pre-hospital critical care from scene to hospital admission for these patients. Despite this, one in ten patients in both groups were hypotensive at arrival in hospital. This is a relatively high number and should warrant further investigation to identify if there are reversible causes that can be corrected to improve haemodynamics before arrival in hospital.

### Decreased level of consciousness

Patients with severe TBI and patients with decreased level of consciousness are at high risk of airway obstruction and hypoxia on-scene due to loss of protective airway reflexes and aspiration of blood and gastric contents [14]. Decreased level of consciousness was the main indication for TI in nearly two thirds of the trauma and non-trauma patients in our study, showing that these patients are an important advanced airway indication group in pre-hospital critical care. Competent airway management is vital in preventing secondary insults and improving outcome in trauma and non-trauma patients with decreased level of consciousness [14, 41]. In our study, the rates of hypoxia and hypotension decreased after TI, and there was little difference between the groups studied from TI to hospital admission.

In trauma patients a GCS score below nine is generally considered as an absolute indication for TI, especially in isolated brain injury [42]. In non-trauma patients however, a GCS score cannot be applied in the same way to support the decision to intubate or not [43]. Decreased level of consciousness must therefore be used in a broader context to support decision-making, e.g. when accompanied by persistent hypoxia despite supplemental oxygen administration [42, 43]. However, some trauma and non-trauma patients with higher GCS scores may benefit from pre-hospital TI to maintain adequate oxygenation and ventilation [44]. A reduction in mortality in patients with GCS below nine receiving pre-hospital physician-led care (instead of paramedic-provided pre-hospital TI) has been shown earlier [45, 46]. A recent review addressing the effect of pre-hospital TI on mortality in patients with severe TBI found a clear trend towards survival when highly trained providers performed TI compared to providers with limited experience [14]. We found that survival to hospital for patients intubated due to decreased level of consciousness was more favourable than for other indications for TI in physician-staffed HEMS. This effect was strong for non-trauma patients, although they presented with lower mean GCS, higher mean age, and had a higher degree of comobidity than trauma patients.

### Survival

In trauma, there is still a high number of potentially preventable deaths on-scene [47]. Massive haemorrhage and non-compressible haemorrhage are important causes of preventable pre-hospital trauma deaths [48, 49]. In both trauma and non-trauma patients, there may be patient-related factors (e.g. cardiopulmonary instability or preexisting comorbidities) that may contribute to lower survival [1]. We have previously shown that pre-hospital TI is safe, with few complications, in the hands of HEMS physicians [24]. In the current study, short-term survival to hospital was not significantly different between trauma and non-trauma patients, and the majority of patients requiring pre-hospital emergency anaesthesia and TI by physician-staffed HEMS presented at the hospital alive.

### Limitations

Although a randomised controlled trial including a control group would have been the preferable standard, this was not feasable in our pre-hospital study setting. The study was therefore designed from a methodological and practical view as a prospective multicenter observational study. Study inclusion was limited to patients who received TI. Those patients for whom TI was attempted but failed and SAD provided were excluded. The data was re-analyzed with these patients included and the results were the same. The strength of this study is the prospective design and the use of a uniform template for data reporting across international HEMS systems. Standardised variables can enhance the quality of data reported, allowing high-quality research data to be compared across patient populations [50]. We believe our results may be generalised to other physician-staffed HEMS. The main limitation was the lack of in-hospital outcome and survival data, which was beyond the scope of this study. Also, the treating physicians recorded the data themselves, with the risk of registration or recall bias. Using anonymous case report forms in this study may have reduced this effect. Automated data capture was not available in the pre-hospital study setting, and physiological data collected at key intervals according to template definitions may not capture all changes in patient physiology.

## Conclusions

Our results showed that non-trauma patients had a higher incidence of hypoxia before TI than trauma patients, but few were hypoxic at admission. The difference for hypotension was less prominent, but one in ten patients were still hypotensive at admission. Further investigations are needed to identify reversible causes that may be corrected to improve haemodynamics in the pre-hospital setting. We found high survival rates to hospital in both groups, suggesting that physician-staffed HEMS provide high-quality emergency airway management in trauma and non-trauma patients.

## Abbreviations

ASA-PS: American Society of Anesthesiologists Physical Status
BVM: Bag-valve-mask ventilation
CI: Confidence interval
cOR: Conditional Odds Ratio
CPAP: Continous positive airway pressure
EMS: Emergency medical services
GCS: Glasgow Coma Score
HEMS: Helicopter Emergency Medical Services
RSI: Rapid sequence intubation
SAD: Supraglottic airway devices
SBP: Systolic blood pressure
SpO_2_: Oxygen saturation
TBI: Traumatic brain injury
TI: Tracheal intubation
